# Supplementary material for: Thalamic interictal epileptic and non-epileptic events during NREM sleep in patients with focal epilepsy: a Stereo-EEG study
Source: eBioMedicine. 2026 May 12;128:106287. doi: 10.1016/j.ebiom.2026.106287 (PMC13193782; doi:10.1016/j.ebiom.2026.106287)
Supplement: Supplementary Materials [file mmc1.docx]

**Table S1. Demographic and clinical data of patients**

| Patient No. | Biological sex | Age, Y | Epilepsy duration, Y | Seizure onset zone | Sampled nuclei | Histopathology | Seizure outcome / Follow up, M |
| --- | --- | --- | --- | --- | --- | --- | --- |
| ZJUT001 | Female | 39.4 | 18.4 | Lt T, P, O | CM, VL, MD | Gliosis | Engel IIb / 35.5 |
| ZJUT002 | Male | 13.8 | 6.8 | Rt F | CM, VL | FCD IIb | Engel IIb / 20.2 |
| ZJUT003 | Male | 33.1 | 6.1 | Lt P | CM, VL | FCD IIb | Engel IIIa / 34.3 |
| ZJUT004 | Female | 26.3 | 13.3 | Lt T | CM, VL | FCD IIIa | Engel IIb / 35.0 |
| ZJUT005 | Female | 29.4 | 17.4 | Lt T | CM, VL | N/A | Engel IVb / 35.8 |
| ZJUT006 | Male | 31.7 | 10.7 | Rt I | CM, VL | Gliosis | Engel IVb / 34.1 |
| ZJUT007 | Male | 21.7 | 16.0 | Rt F | CM, VL | FCD Ib | Engel Ia / 33.2 |
| ZJUT008 | Male | 26.8 | 2.5 | Rt F | CM, VL | FCD IIa | Engel Ia / 33.4 |
| ZJUT009 | Male | 25.4 | 12.4 | Diffuse | CM, VL | N/A | Surgically non-remediable |
| ZJUT010 | Female | 7.2 | 2.2 | Diffuse | CM, VL, Pf | N/A | Surgically non-remediable |
| ZJUT011 | Female | 19.3 | 10.3 | Rt MTL | CM, VL | HS | Engel Ia / 32.0 |
| ZJUT012 | Male | 17.2 | 5.2 | Diffuse | CM, VL | N/A | Surgically non-remediable |
| ZJUT014 | Male | 10.5 | 1.8 | Rt F | Pu, VL | FCD IIa | Engel IVb / 26.7 |
| ZJUT015 | Female | 19.6 | 12.6 | Lt F | CM, VL | TSC | Engel Ia / 26.8 |
| ZJUT016 | Male | 9.3 | 3.3 | Lt MTL | CM, VL | Non-specific | Engel Ia / 29.4 |
| ZJUT017 | Male | 16.9 | 7.9 | Lt P | CM, VL | FCD I | Engel IVa / 29.0 |
| ZJUT018 | Male | 39.9 | 27.9 | Lt MTL; Rt MTL | CM, VPL | N/A | Surgically non-remediable |
| ZJUT019 | Female | 31.1 | 10.1 | Lt MTL | CM, VL | Gliosis | Engel Ia / 28.3 |
| ZJUT020 | Female | 26.9 | 25.2 | Rt MTL | CM, VL | Gliosis | Engel Ib / 26.7 |
| ZJUT021 | Male | 34.0 | 32.0 | Lt MTL | CM, VL, Pf | HS | Engel Ia / 26.7 |
| ZJUT022 | Male | 27.1 | 4.2 | Lt MTL; Rt MTL | CM, VL | N/A | Surgically non-remediable |
| ZJUT023 | Female | 18.9 | 10.9 | Diffuse | CM, VL | N/A | Surgically non-remediable |
| ZJUT024 | Male | 11.7 | 4.0 | Rt T | CM, VPL | Gliosis | Engel IVb / 26.9 |
| ZJUT025 | Female | 13.1 | 7.1 | Rt F | CM, VPL | Gliosis | Engel Ia / 21.6 |
| ZJUT026 | Male | 25.4 | 13.4 | Rt, T, P, O | CM, VPL | N/A | Engel IIIa / 24.5 |
| ZJUT027 | Male | 9.7 | 8.7 | Rt I | CM, VPL | FCD IIa | Engel IVb / 23.4 |
| ZJUT028 | Male | 15.8 | 8.8 | Rt T, P, O | CM, VPL, Pf | FCD Ia | Engel Ib / 24.1 |
| ZJUT030 | Female | 22.7 | 7.7 | Rt T, P, O | CM, VPL | FCD Ia | Engel IVb / 22.0 |
| ZJUT031 | Female | 33.1 | 15.1 | Lt F | CM, VL, VPL | FCD IIb | Engel Ia / 22.5 |
| ZJUT032 | Male | 24.7 | 7.7 | Rt T, P, O | CM, VL, VPL | Gliosis | Engel Ib / 21.1 |
| ZJUT033 | Male | 19.3 | 14.3 | Lt F | CM, VL, VPL | Gliosis | Engel IIb / 20.9 |
| ZJUT034 | Female | 18.4 | 5.4 | Lt I | CM, VPL | FCD II | Engel Ia / 20.6 |
| ZJUT035 | Male | 15.6 | 7.6 | Lt MTL, I | CM, VPL | Gliosis | Engel Ia / 19.7 |
| ZJUT037 | Male | 25.4 | 22.4 | Lt I | CM, VL | Gliosis | Engel IIIa / 19.5 |
| ZJUT038 | Female | 19.0 | 11.0 | Lt T, P, O | CM, VL, VPL | Non-specific | Engel IVb / 20.0 |
| ZJUT039 | Male | 20.7 | 7.7 | Lt T | CM, VL | Gliosis | Engel Ib / 19.3 |
| ZJUT040 | Female | 27.5 | 23.5 | Lt F, T | CM, VL | N/A | Surgically non-remediable |
| ZJUT041 | Female | 31.1 | 18.6 | Lt T, P, O | CM, VL | N/A | Surgically non-remediable |
| ZJUT042 | Male | 45.8 | 33.8 | Diffuse | CM, VL | N/A | Surgically non-remediable |
| ZJUT043 | Male | 29.2 | 10.2 | Lt T | VL, MD | N/A | Engel Ia / 18.5 |
| ZJUT044 | Male | 16.3 | 11.3 | Lt F | Pu, VPL | N/A | Engel Ia / 16.5 |
| ZJUT045 | Female | 11.6 | 6.6 | Rt P | CM, VPL | Tumor | Engel Ia / 15.4 |
| ZJUT046 | Female | 27.6 | 12.6 | Lt T, O | CM, VPL | Heterotopia | Engel IIb / 12.0 |
| ZJUT047 | Female | 25.4 | 24.4 | Rt P | VPL | N/A | N/A |
| ZJUT048 | Female | 33.6 | 9.6 | Lt MTL | CM, VL, VPL | Gliosis | Engel IIa / 15.1 |
| ZJUT049 | Female | 33.4 | 10.4 | Rt F | VPL | Gliosis | Engel IVa / 14.0 |
| ZJUT051 | Male | 31.2 | 10.2 | Rt MTL | CM, VPL | N/A | N/A |
| DEP00003 | Female | 23.0 | 18.0 | Lt T | CM, VL, VPL | Gliosis | Engel IIb / 22.0 |
| DEP00005 | Female | 34.0 | 28.0 | Rt F, T | CM, Pu, VPL | FCD Ib | Engel IIIa / 16.0 |
| DEP00010 | Female | 19.0 | 2.0 | Diffuse | Pu, VPL | N/A | Surgically non-remediable |
| DEP00011 | Male | 30.0 | 25.0 | Rt T | Pu, VL, VPL | Non-specific | Engel Ia / 18.0 |
| DEP00020 | Female | 23.0 | 17.0 | Lt O | Pu | N/A | Surgically non-remediable |
| DEP00032 | Male | 38.0 | 10.0 | Lt T | Pu | N/A | Surgically non-remediable |
| DEP00041 | Female | 59.0 | 49.0 | Lt MTL | CM, VPL | N/A | Engel Ia / 18.0 |
| DEP00042 | Male | 18.0 | 8.0 | Lt T | CM, VPL | Non-specific | Engel IVb / 16.0 |
| DEP00062 | Male | 16.0 | 13.0 | Lt T | CM, VL, LGN | Gliosis | Engel Ia / 12.0 |
| DEP00067 | Female | 28.0 | 19.0 | Lt F, T | Pu, VPL | N/A | Surgically non-remediable |
| DEP00068 | Male | 24.0 | 13.0 | Lt T | CM, VPL | FCD Ic | Engel IIb / 12.0 |
| DEP00069 | Female | 25.0 | 21.0 | Lt O | Pu | N/A | Engel IIb / 12.0 |
| DEP00070 | Female | 41.0 | 13.0 | Lt MTL | Pu | Gliosis | Engel Ia / 12.0 |
| DEP00095 | Female | 30.0 | 24.0 | Lt F, T | CM, VL, VPL | N/A | Surgically non-remediable |
| DEP00101 | Female | 46.0 | 33.0 | Rt F | VPL | N/A | N/A |
| DEP00105 | Male | 45.0 | 28.0 | Lt T; Rt T | CM, VPL | N/A | Surgically non-remediable |
| DEP00108 | Female | 44.0 | 29.0 | Lt MTL; Rt T | Pu | N/A | Surgically non-remediable |

Y, years; M, months; Rt, right; Lt, left; F, frontal lobe; I, insular; O, occipital lobe; P, parietal lobe; T, temporal lobe; MTL, mesial temporal lobe; CM, centromedian nucleus; VL, ventral lateral nucleus; VPL, ventral posterolateral nucleus; MD, mediodorsal nucleus; Pf, parafascicular nucleus; Pu, pulvinar; LGN, lateral geniculate nucleus; HS, hippocampal sclerosis; FCD, focal cortical dysplasia; N/A, not available; TSC, tuberous sclerosis.

**Table S2 Subregions of the sampled Pulvinar**

| **Patient No.** | **Subregions** |
| --- | --- |
| ZJUT014 | Medial Pulvinar |
| ZJUT044 | Anterior Pulvinar |
| DEP00005 | Anterior Pulvinar |
| DEP00010 | Medial and Inferior Pulvinar |
| DEP00011 | Anterior Pulvinar |
| DEP00020 | Medial Pulvinar |
| DEP00032 | Medial and Inferior Pulvinar |
| DEP00067 | Inferior Pulvinar |
| DEP00069 | Medial Pulvinar |
| DEP00070 | Medial Pulvinar |
| DEP00108 | Medial and Inferior Pulvinar |

**Table S3. Interictal event detectors**

| **Detectors** | **Links** | **References** |
| --- | --- | --- |
| Spindle | N/A | (Mölle et al., 2011; Schiller et al., 2025) |
| Spike | <https://github.com/EpiReC-ISARG/IED_detector> | (Janca et al., 2015) |
| Spike-gamma | <https://doi.org/10.5281/ZENODO.11237651> | (Thomas et al., 2022) |
| HFO | <https://mni-open-ieegatlas.research.mcgill.ca/> | (von Ellenrieder et al., 2012) |

**Table S4. Multivariate analysis of the correlation between thalamic spike-fast activity and demographic factors on seizure outcomes**

| **Nuclei** | **Comparisons** | **Variates** | **Odds Ratio** | **95% CI** | ***p* value** |
| --- | --- | --- | --- | --- | --- |
| **Th** | Engel I  vs.  Others | Age | 1.03 | 0.95 – 1.14 | 0.47 |
|  |  | Duration | 0.97 | 0.88 – 1.07 | 0.54 |
|  |  | Sex category | 1.03 | 0.31 – 3.50 | 0.96 |
|  |  | Spike-fast activity | **1.57** | 1.21 – 2.13 | 0.002 |
|  | Engel I  vs.  Engel II-IV | Age | 1.02 | 0.92 – 1.12 | 0.72 |
|  |  | Duration | 0.96 | 0.87 – 1.07 | 0.51 |
|  |  | Sex category | 1.03 | 0.28 – 3.73 | 0.97 |
|  |  | Spike-fast activity | **1.48** | 1.10 – 1.99 | 0.01 |
|  | Engel I  vs.  Surgically non-remediable | Age | 1.06 | 0.95 – 1.18 | 0.28 |
|  |  | Duration | 0.97 | 0.86 – 1.10 | 0.86 |
|  |  | Sex category | 1.01 | 0.22 – 4.56 | 0.99 |
|  |  | Spike-fast activity | **1.77** | 1.19 – 2.64 | 0.005 |
| **CM** | Engel I  vs.  Others | Age | 1.05 | 0.95 – 1.17 | 0.33 |
|  |  | Duration | 1.00 | 0.88 – 1.12 | 0.94 |
|  |  | Sex category | 1.15 | 0.32 – 4.14 | 0.83 |
|  |  | Spike-fast activity | **1.42** | 1.08 – 1.96 | 0.02 |
|  | Engel I  vs.  Engel II-IV | Age | 1.05 | 0.94 – 1.17 | 0.38 |
|  |  | Duration | 0.98 | 0.86 – 1.11 | 0.71 |
|  |  | Sex category | 1.03 | 0.25 – 4.17 | 0.97 |
|  |  | Spike-fast activity | **1.47** | 1.06 – 2.04 | 0.02 |
|  | Engel I  vs.  Surgically non-remediable | Age | 1.06 | 0.95 – 1.17 | 0.40 |
|  |  | Duration | 1.02 | 0.88 – 1.12 | 0.79 |
|  |  | Sex category | 1.30 | 0.32 – 4.14 | 0.75 |
|  |  | Spike-fast activity | 1.34 | 1.08 – 1.96 | 0.11 |
| **VL** | Engel I  vs.  Others | Age | 1.02 | 0.90 – 1.16 | 0.75 |
|  |  | Duration | 0.97 | 0.84 – 1.10 | 0.63 |
|  |  | Sex category | 0.62 | 0.11 – 3.24 | 0.57 |
|  |  | Spike-fast activity | **1.73** | 1.22 – 2.63 | 0.004 |
|  | Engel I  vs.  Engel II-IV | Age | 1.02 | 0.90 – 1.16 | 0.73 |
|  |  | Duration | 0.95 | 0.83 – 1.09 | 0.49 |
|  |  | Sex category | 0.65 | 0.11 – 3.72 | 0.63 |
|  |  | Spike-fast activity | **1.56** | 1.05 – 2.32 | 0.03 |
|  | Engel I  vs.  Surgically non-remediable | Age | 1.02 | 0.87 – 1.19 | 0.82 |
|  |  | Duration | 1.00 | 0.85 – 1.17 | 0.99 |
|  |  | Sex category | 0.56 | 0.07 – 4.50 | 0.59 |
|  |  | Spike-fast activity | **2.17** | 1.25 – 3.77 | 0.006 |

This generalised linear model included spike-fast activity rate, age, biological sex, and epilepsy duration as variables. An odds ratio less than zero indicates that the outcome is more likely to be of Engel I. *n_total_* = 60; Th, thalamus as a whole; CM, centromedian nucleus; VL, ventral lateral nucleus. Others: Engel II-IV and surgically non-remediable groups; The unit for age and epilepsy duration: years; The unit for spike-fast activity: logarithmic occurrence rates (/min).

**Table S5. Median frequency and duration of thalamic ripples and fast ripples across different nuclei**

| **Nuclei** | **Ripple** | | **Fast ripple** | |
| --- | --- | --- | --- | --- |
|  | Frequency (Hz) | Duration (ms) | Frequency (Hz) | Duration (ms) |
| Entire thalamus | 124.37 | 62.00 | 355.00 | 26.00 |
| CM | 150.00 | 53.00 | 307.50 | 28.50 |
| VL | 112.50 | 69.87 | 381.25 | 25.00 |
| VPL | 110.00 | 62.75 | 394.37 | 24.50 |
| Pu | 115.00 | 66.75 | 355.00 | 26.50 |

CM, centromedian nucleus, *n_total_* = 50; VL, ventral lateral nucleus, *n_total_* = 36; VPL, ventral posterolateral nucleus, *n_total_* = 31; Pu, pulvinar, *n_total_* = 11.


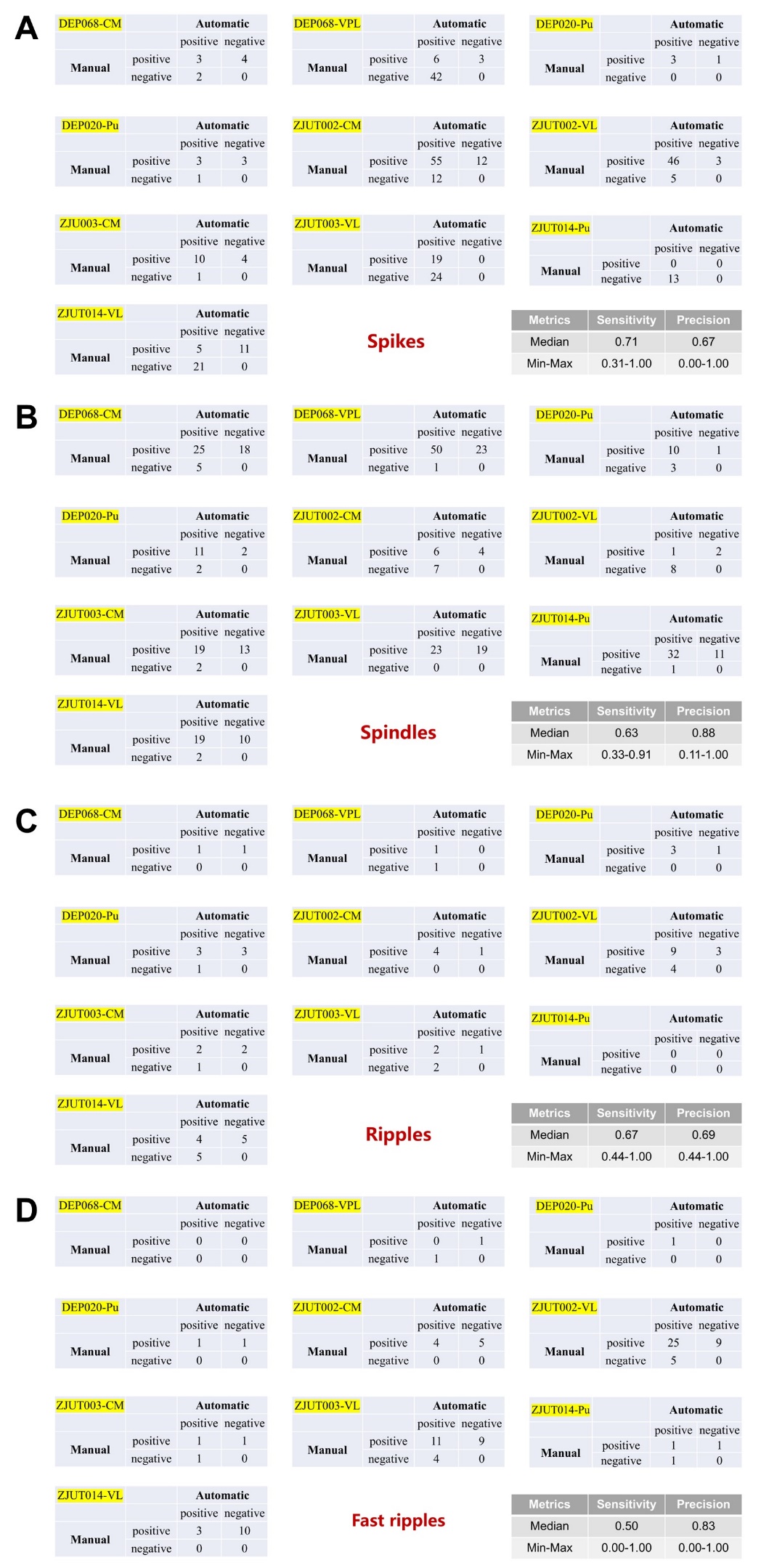


**Figure S1**. Performance of interictal transient event detectors based on 5-minute thalamic recordings. The automatically detected markers were labeled as either positive or negative according to the manual annotations. The upper left corner of the table shows the subject number and the thalamic nucleus in which the bipolar channel is located. Sensitivity and precision metrics are presented in the lower right corner of each figure. CM, centromedian nucleus; Pu, pulvinar nuclei; VL, ventral lateral nucleus; VPL, ventral posterolateral nucleus.


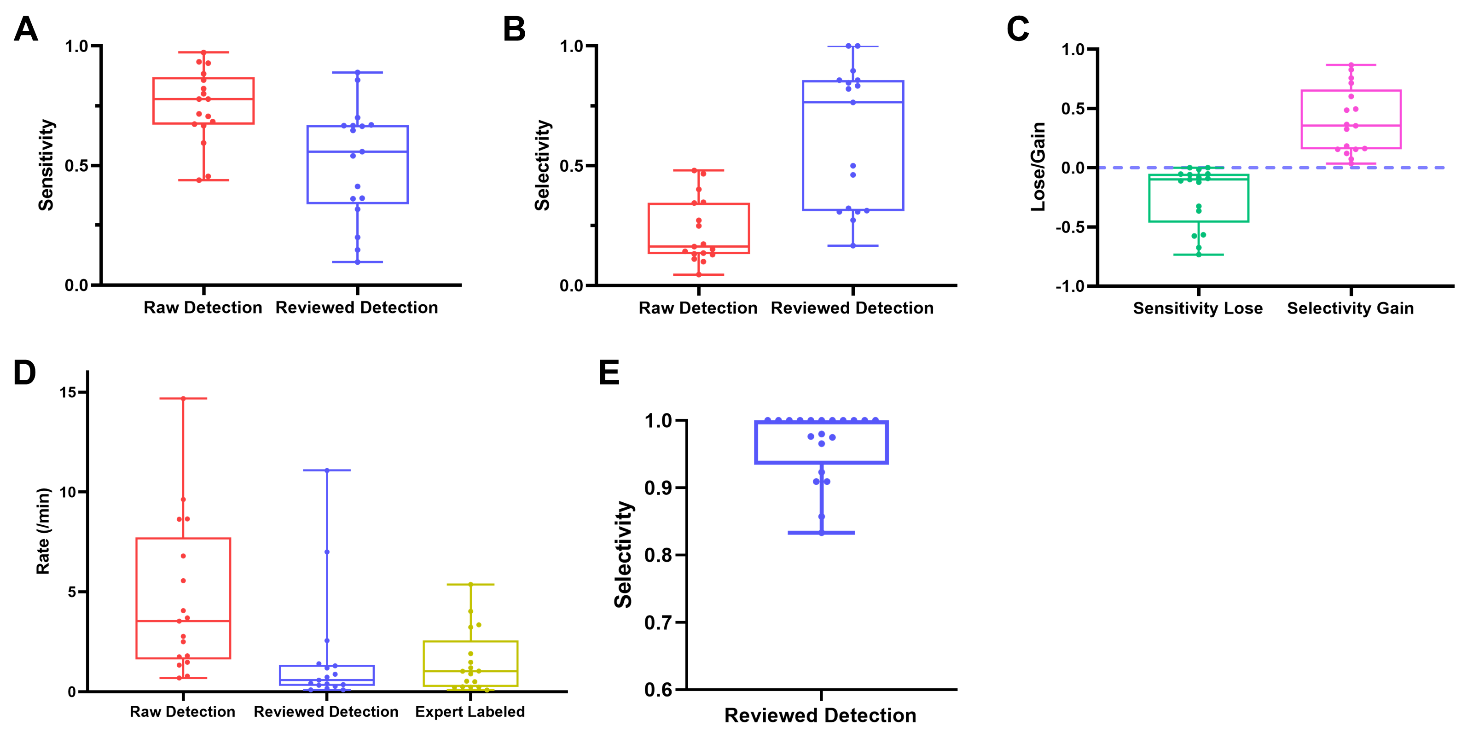


**Figure S2**. Two-round validation of the semi-automated thalamic spike detection pipeline. To evaluate the trade-off between sensitivity and selectivity, a two-round validation was performed on a subset of ten patients. Round 1: A board-certified epileptologist (I.D.) manually annotated all spikes and sharp waves (20–200 ms) to serve as the Gold Standard. A and B show the sensitivity and selectivity results, respectively; C shows the selectivity gain and sensitivity loss of the manual verification procedure; D shows the event rates (events/min) detected by the raw automated detector, the semi-automated pipeline, and the Gold Standard. *n* = 17. Round 2: the expert reviewed all events marked by the semi-automated pipeline in the same subset and marked whether they were true epileptic spikes or sharp waves. E: The selectivity of the detection, *n* = 20. Median values, interquartile ranges, maximum values, and minimum values are shown in the plots. Each dot represents one thalamic channel.


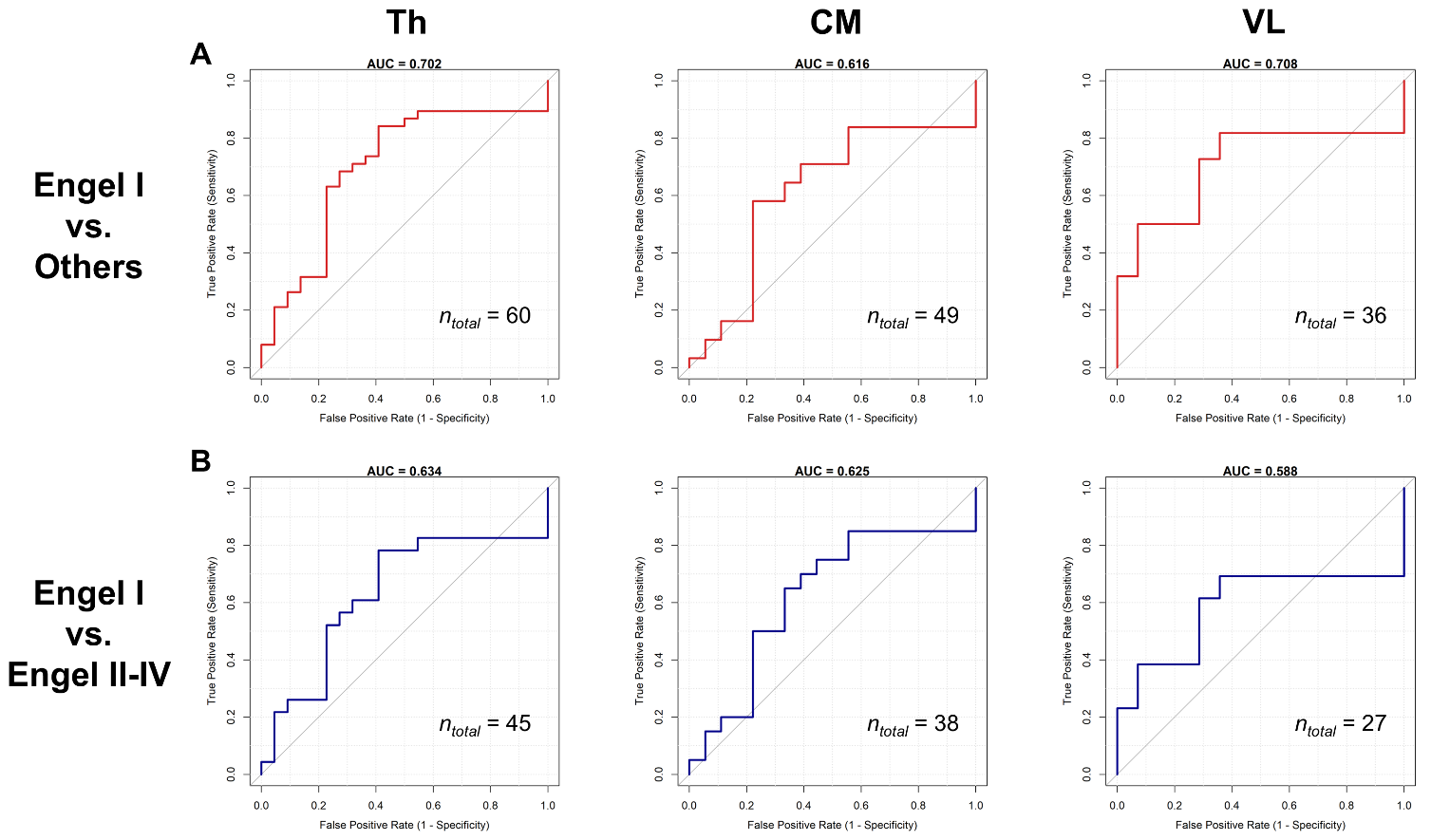


**Figure S3**. Predictive performance of thalamic spike-fast activity using logistic regression and Leave-One-Out Cross-Validation. A: ROC curve distinguishing the Engel I group from others (the combination of Engel II-IV and surgically non-remediable groups). B: ROC curve distinguishing the Engel I group from the Engel II-IV group. The Area Under the Curve (AUC) values are displayed within each plot. The unit for spike-fast activity: logarithmic occurrence rates (/min). ROC: Receiver Operating Characteristic.


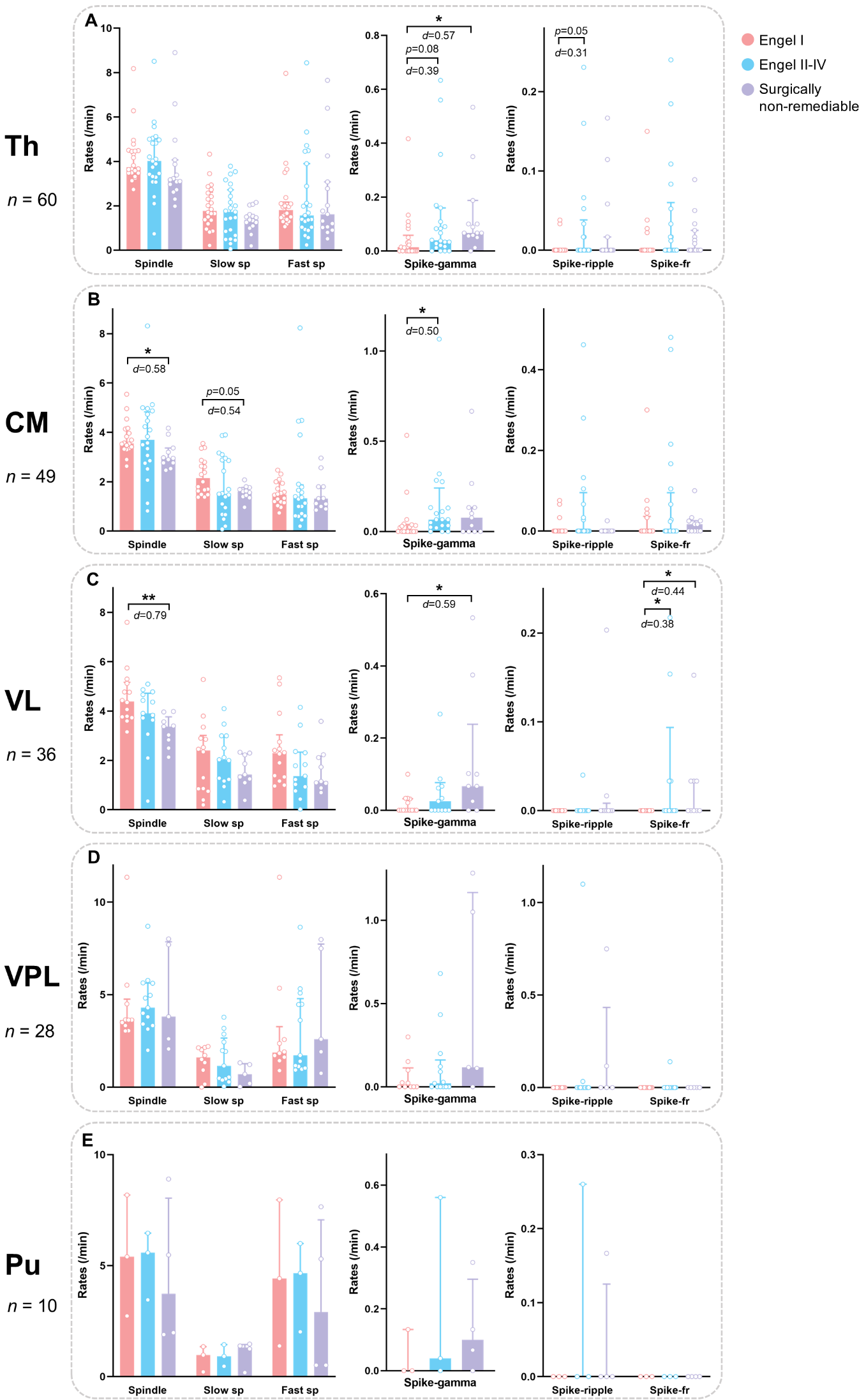


**Figure S4**. Rates of interictal transient events in different thalamic nuclei and in three outcome groups. A: Rates of interictal events in the thalamus as a whole. B–E: Rates of interictal events in the CM, VL, VPL, and Pu, respectively. Median values and interquartile ranges are shown in the plots. Each dot represents one subject. Statistical test: Bonferroni-corrected post-hoc comparisons following Kruskal-Wallis test; *, p < 0.05; **, p < 0.01. Th, thalamus; CM, centromedian nucleus; Pu, pulvinar nuclei; VL, ventral lateral nucleus; VPL, ventral posterolateral nucleus; sp, spindle; fr, fast ripple.


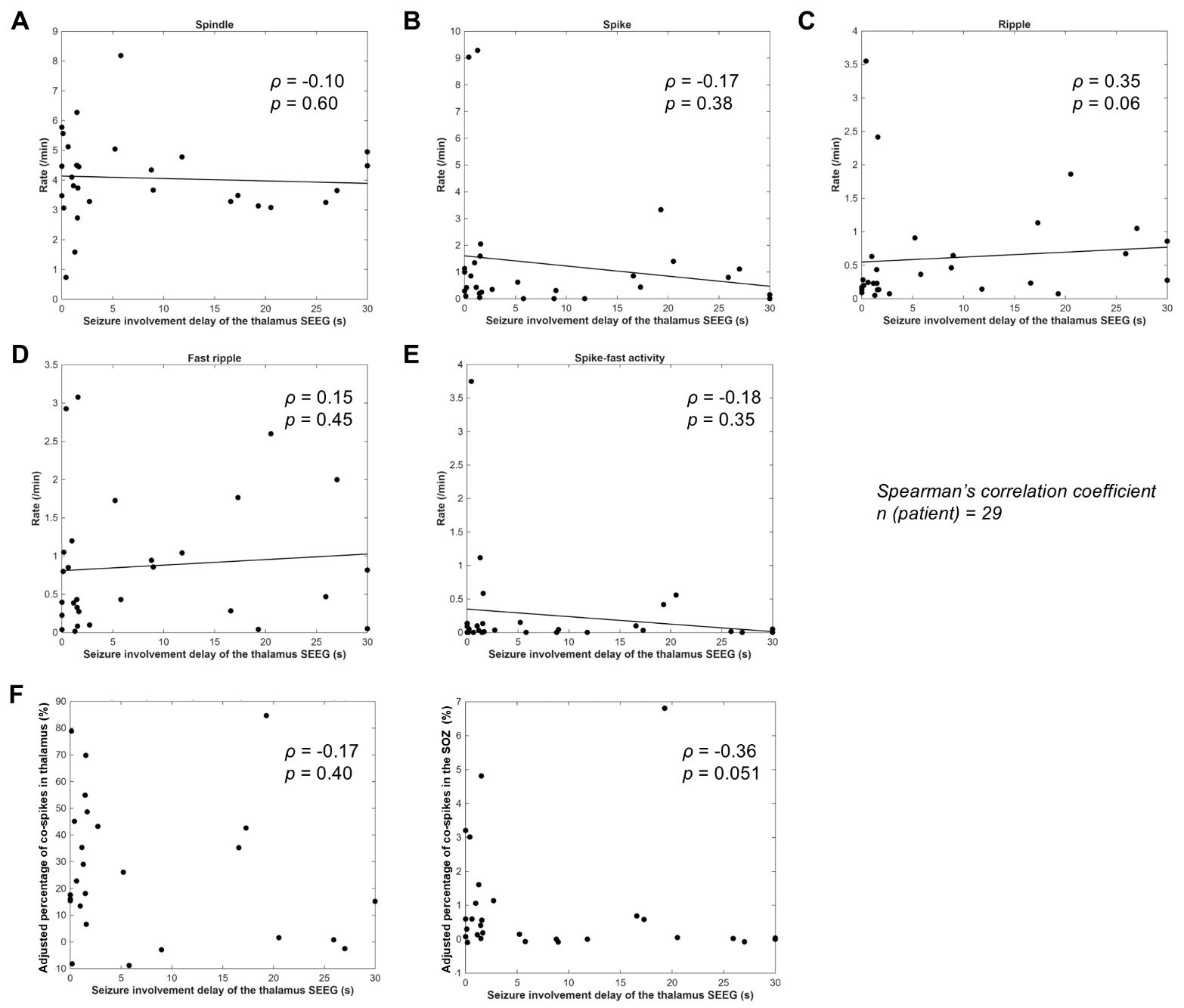


**Figure S5**. Correlation between thalamic ictal involvement and interictal metrics. The analysis was performed on a subset of 29 patients with focal SOZ. The X-axis represents the median delay of thalamic seizure involvement relative to the SOZ; cases with no involvement or delay >30s were censored at 30s. ρ represents the Spearman’s correlation coefficient. A–E: Scatter plots showing the Spearman correlation between thalamic involvement delay and the rates of interictal events. F: Spearman correlation between thalamic ictal involvement delay and the adjusted proportion of SOZ-thalamic co-spikes. SOZ, seizure onset zone; Th, thalamus as a whole; SEEG, stereo-electroencephalography.


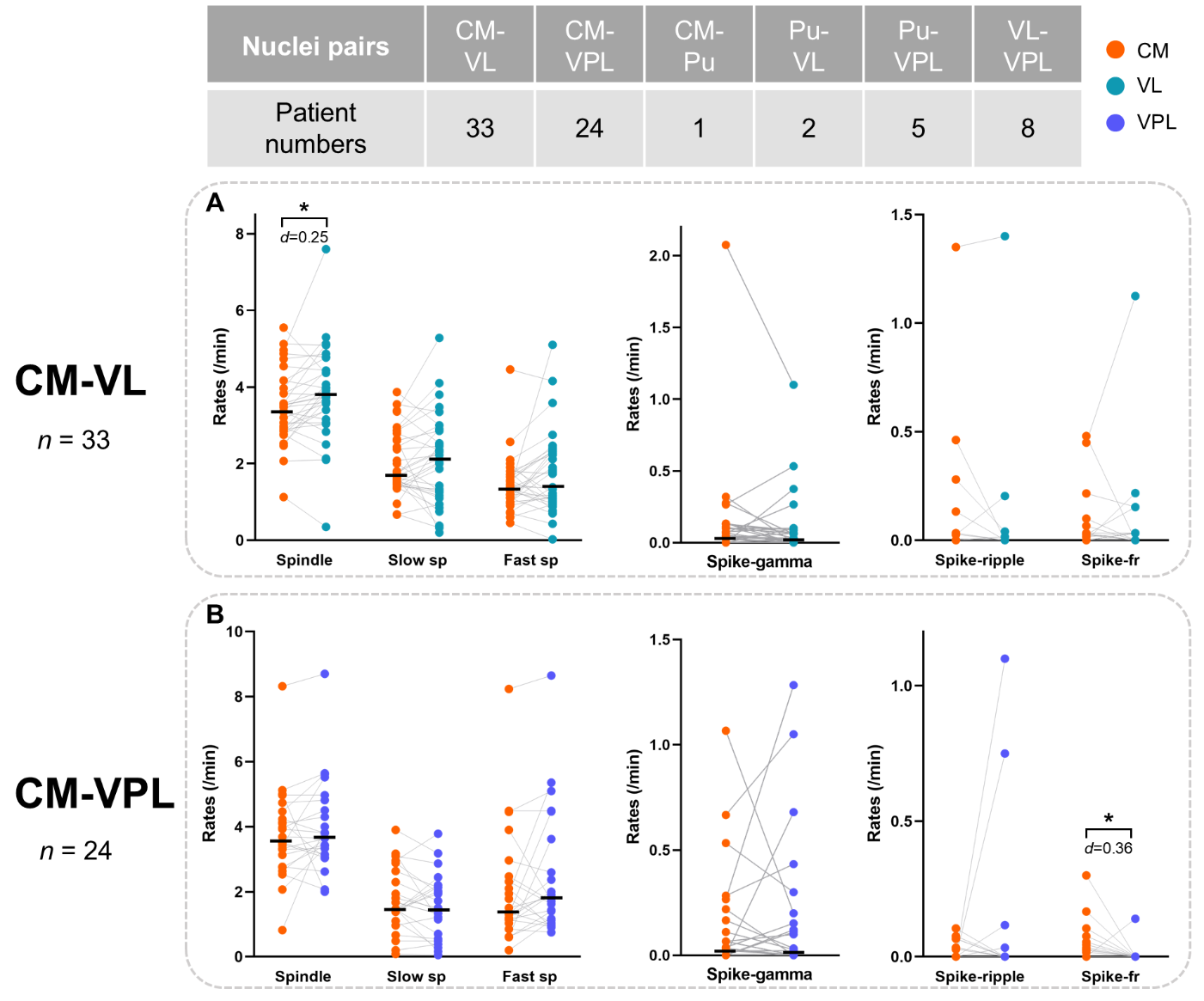


**Figure S6**. Pairwise comparisons of events’ rates between different nuclei. The table summarises the numbers of all the nuclei pairs within a subject. Black lines indicate median values. Each dot represents one subject. Statistical test: Wilcoxon signed-rank test; *, p < 0.05. CM, centromedian nucleus; Pu, pulvinar nuclei; VL, ventral lateral nucleus; VPL, ventral posterolateral nucleus; sp, spindle; fr, fast ripple.


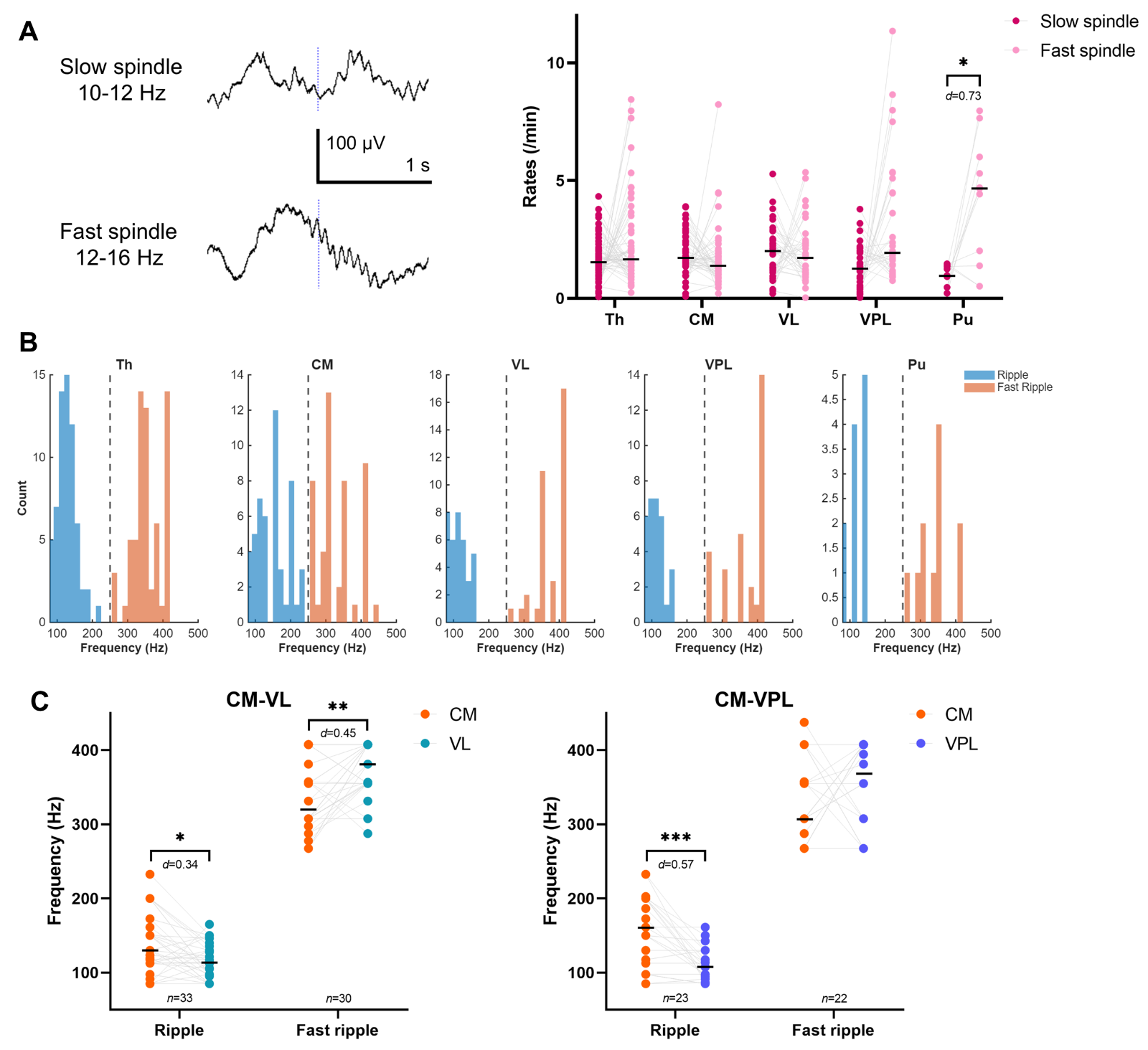


**Figure S7**. Frequency of spindles and HFOs across different thalamic nuclei. A: Examples of slow spindles (10-12 Hz) and fast spindles (12-16 Hz), and the paired comparisons of their rates within individuals. For the Th, CM, VL, VPL, and Pu groups, the sample sizes were 64, 50, 36, 31, and 11, respectively. B: Frequency distribution of thalamic HFOs. The histogram displays the aggregate frequency distribution of ripple and fast ripple events across the patient cohort. The y-axis represents the number of cases, and the gray dashed line indicates the cutoff frequency of 250 Hz. For the Th, CM, VL, VPL, and Pu groups, the sample sizes were 64, 50, 36, 31, and 11, respectively. C: Pairwise comparisons of the median frequency of ripples (80-250 Hz) and fast ripples (250-500 Hz) between different thalamic nuclei within individuals. Black lines indicate median values. Each dot represents one subject. Statistical test: Wilcoxon signed-rank test; *, p < 0.05; **, p < 0.01; ***, p < 0.001. HFOs, high frequency oscillations; Th, thalamus; CM, centromedian nucleus; Pu, pulvinar nuclei; VL, ventral lateral nucleus; VPL, ventral posterolateral nucleus.


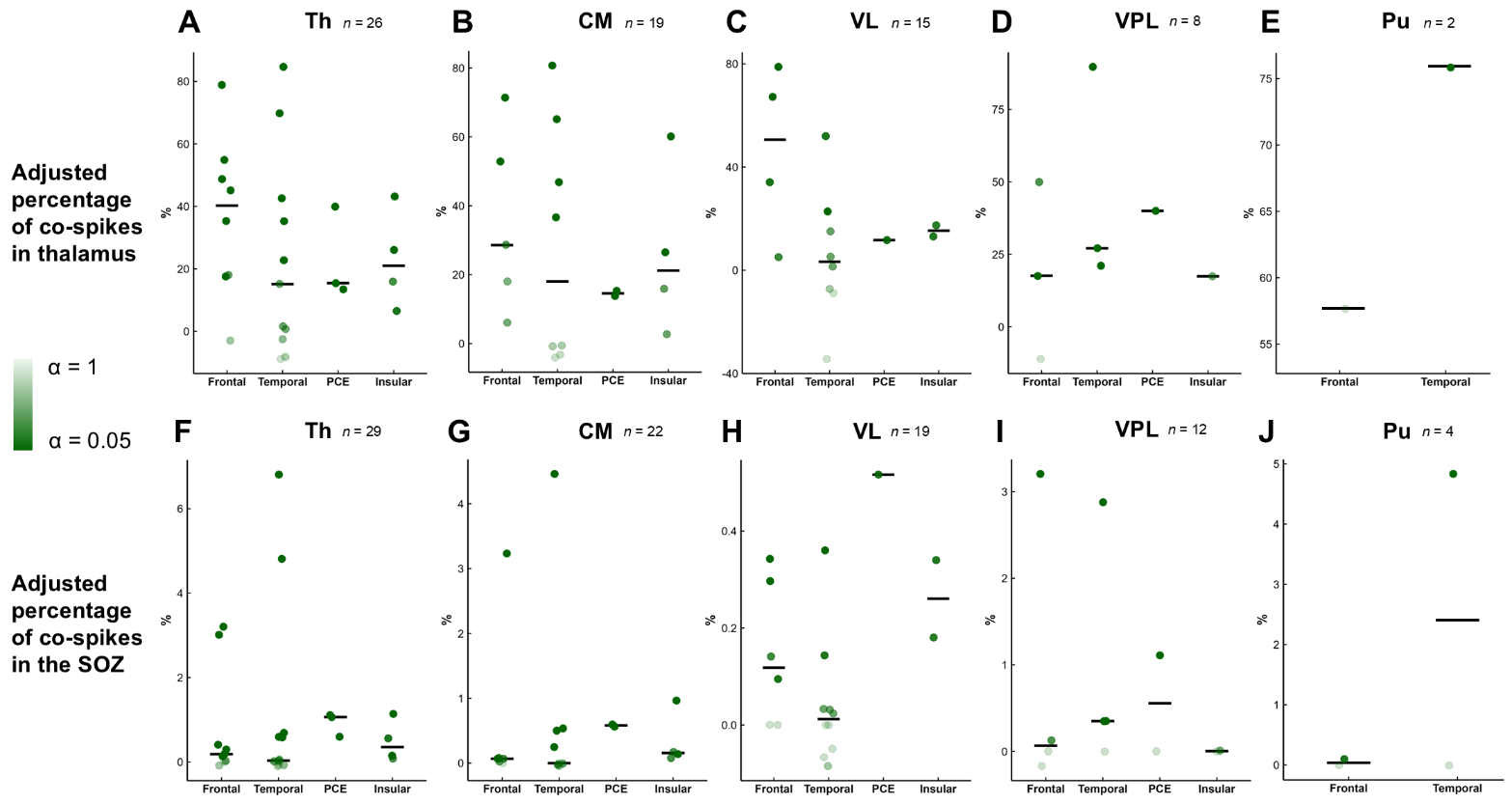


**Figure S8**. Proportion of co-spikes between the SOZ and the thalamus in different epilepsy types. The x-axis represents the type of epilepsy categorised according to the location of the SOZ. Each dot represents one subject. The transparency of the dot colour indicates the alpha value in the surrogate data analysis. Lower transparency indicates that the number of co-spikes is higher than the chance level. Statistical test: non-parametric Kruskal-Wallis test. SOZ, seizure onset zone; PCE, posterior cortex epilepsy. Th, thalamus; CM, centromedian nucleus; Pu, pulvinar nuclei; VL, ventral lateral nucleus; VPL, ventral posterolateral nucleus.


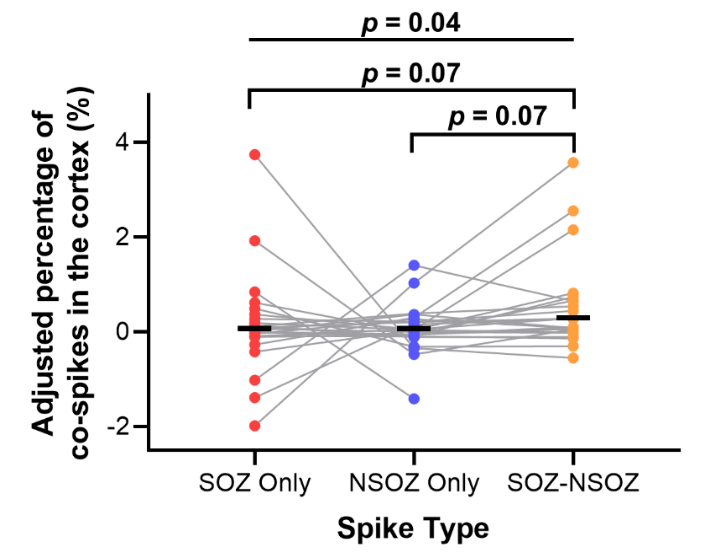


**Figure S9**. The adjusted proportion of co-spikes between cortical and thalamic spikes. Cortical spikes were categorised into three groups: SOZ-only (spikes confined to the SOZ), NSOZ-only (spikes confined to the NSOZ), and SOZ-NSOZ (synchronous spikes involving both zones). Adjusted percentages of co-spikes were derived by using the number of cortical spikes as the denominator. Statistical test: non-parametric Friedman Test with post-hoc comparisons and Bonferroni correction, *n* = 25. SOZ, seizure onset zone; NSOZ, non-SOZ.


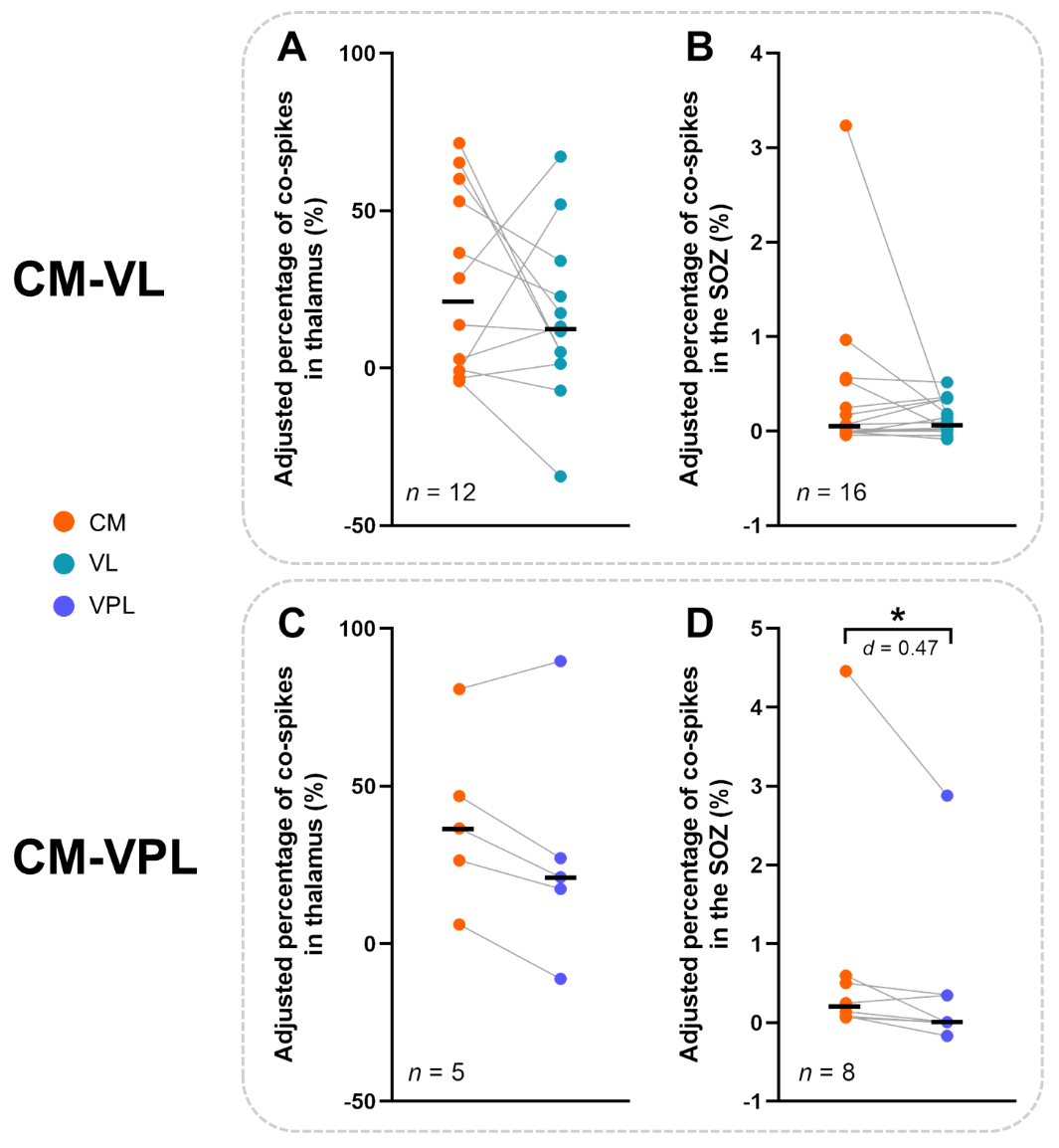


**Figure S10**. Pairwise comparisons of proportions of co-spikes with the SOZ between different nuclei. Percentages of co-spikes were calculated using the number of spikes in the thalamic nuclei and the SOZ as the denominator, respectively. Black lines indicate median values. Each dot represents one subject. Statistical test: Wilcoxon signed-rank test; *, p < 0.05. CM, centromedian nucleus; VL, ventral lateral nucleus; VPL, ventral posterolateral nucleus.


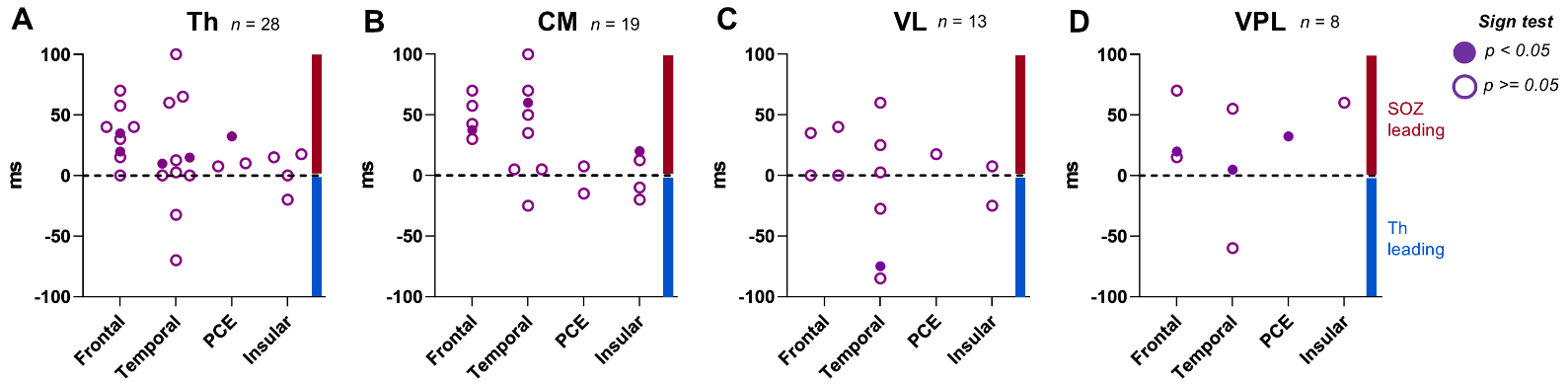


**Figure S11**. The median co-occurrence interval between the seizure onset zone and thalamic spikes. The x-axis represents the type of epilepsy categorised according to the location of the seizure onset zone. Each dot represents one subject. Statistical test: Sign test. The solid dots indicate significant unidirectional spike propagation between the seizure onset zone and the thalamic channel. PCE, posterior cortex epilepsy; Th, thalamus; CM, centromedian nucleus; Pu, pulvinar nuclei; VL, ventral lateral nucleus; VPL, ventral posterolateral nucleus.


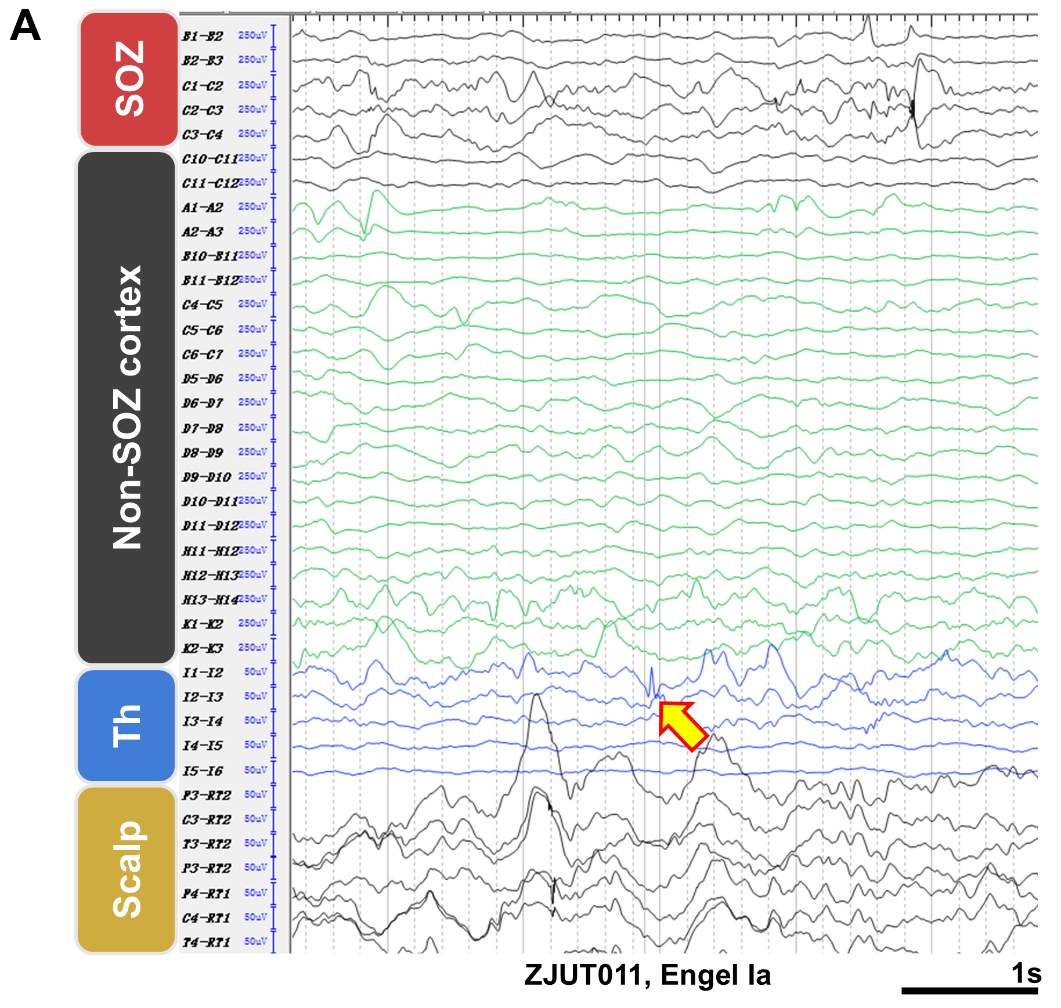


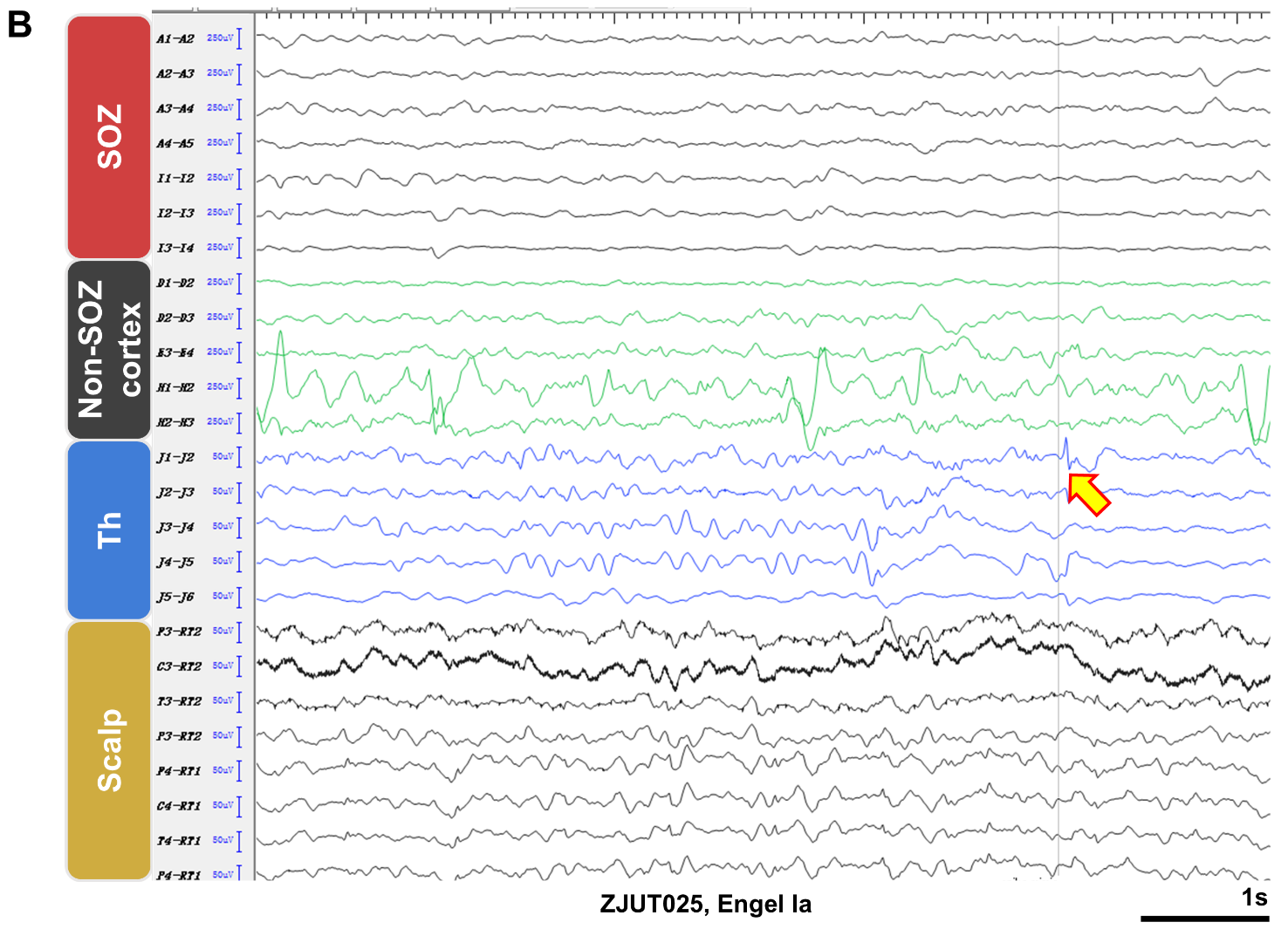


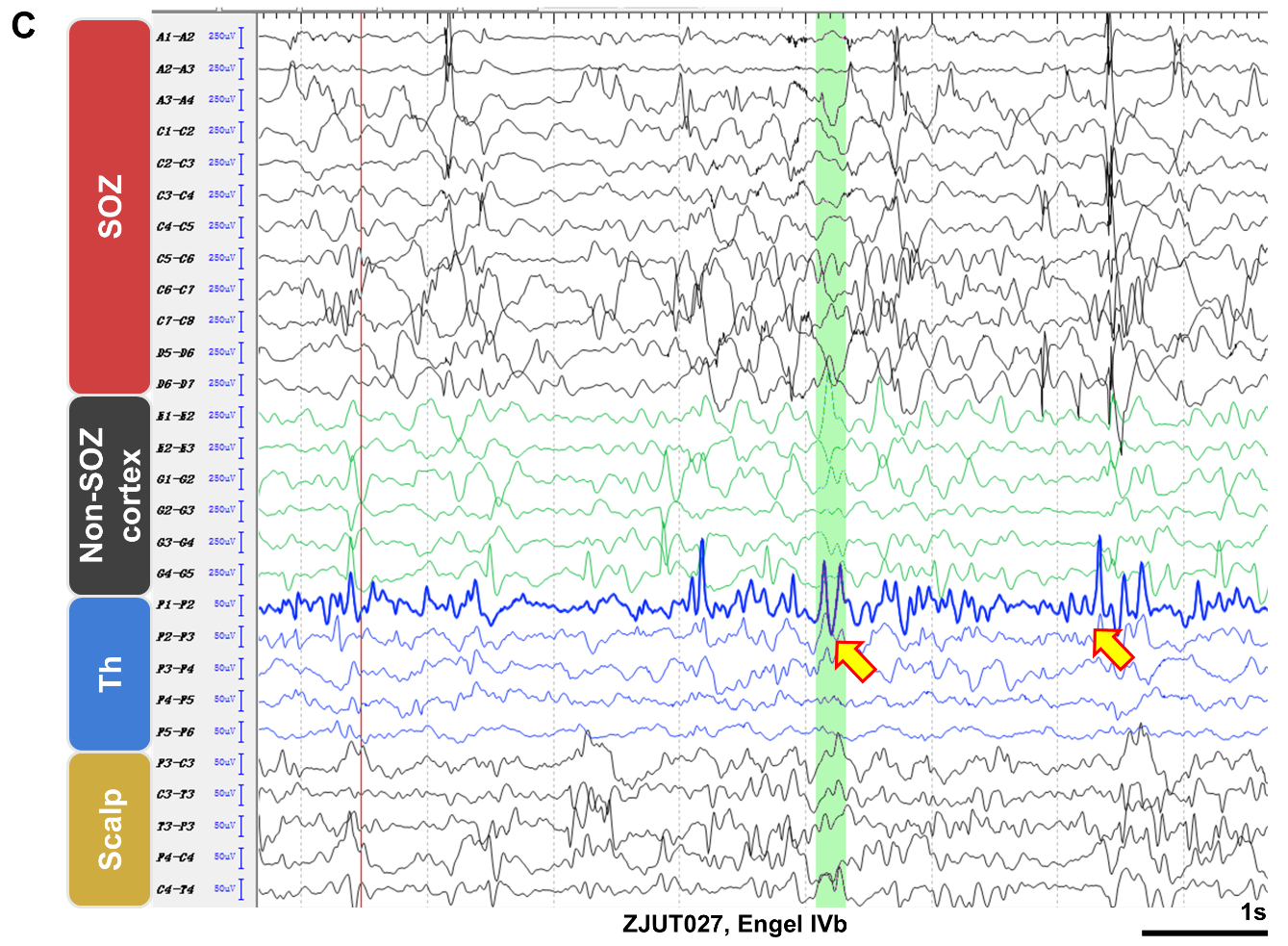


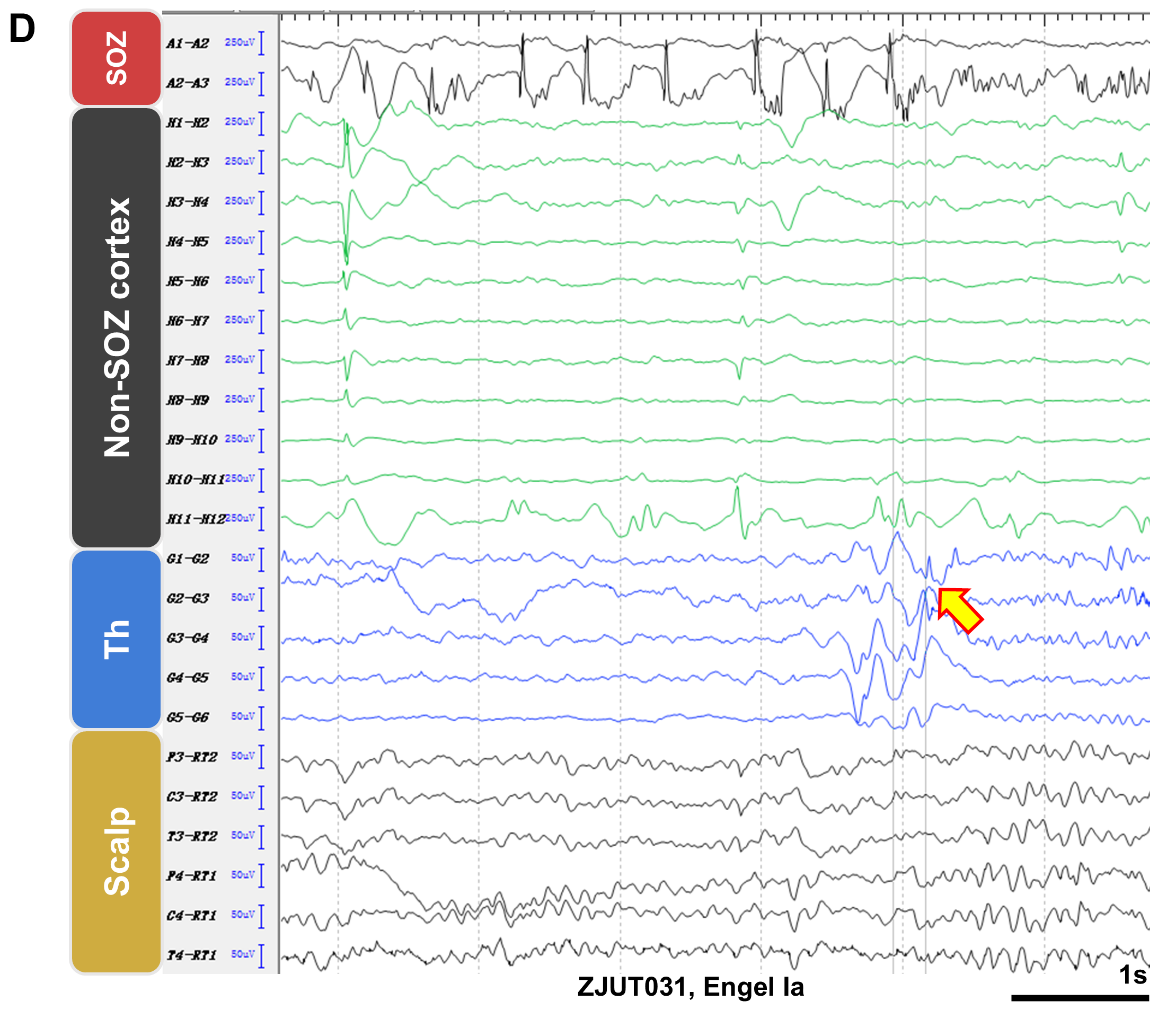


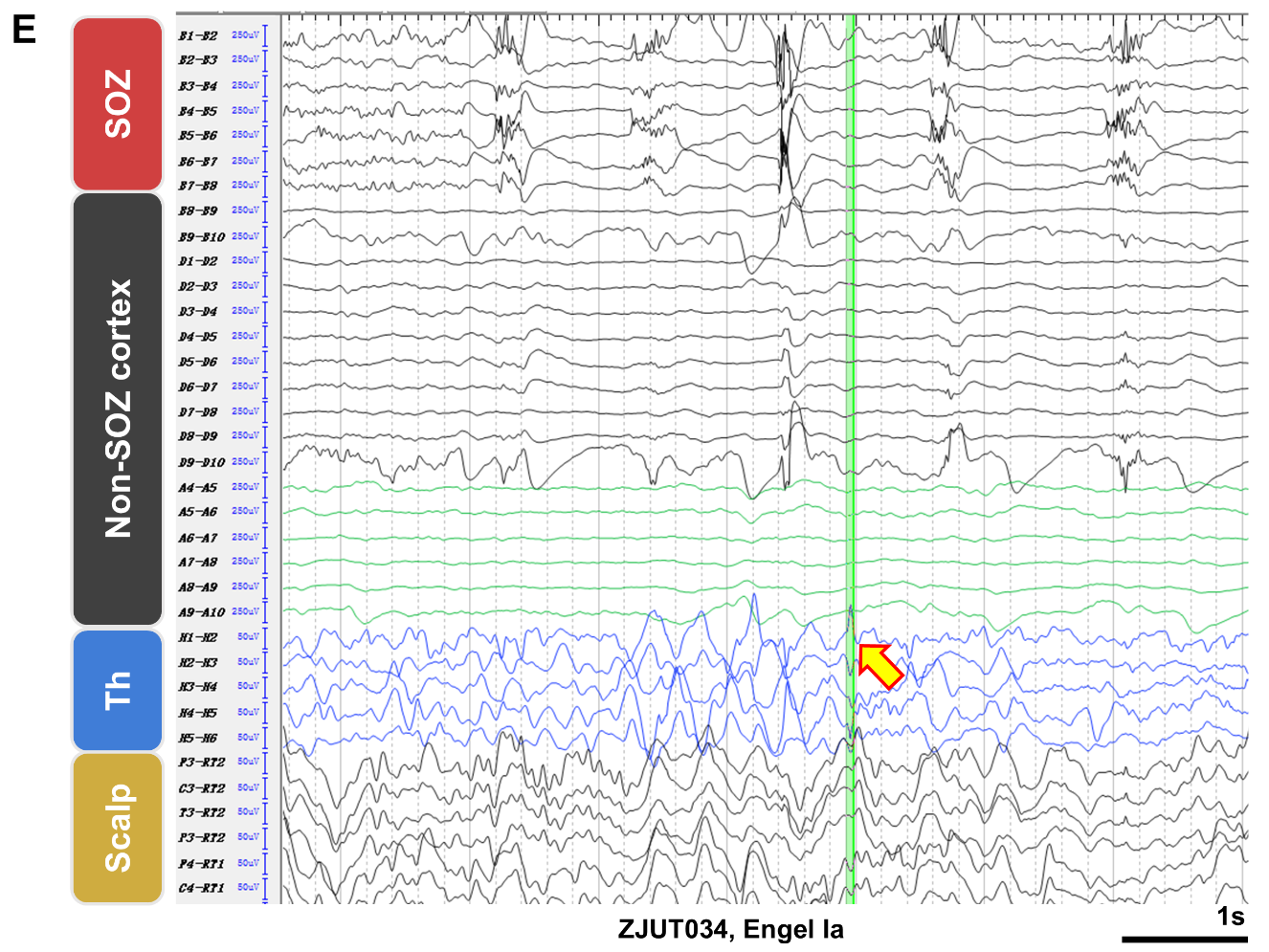


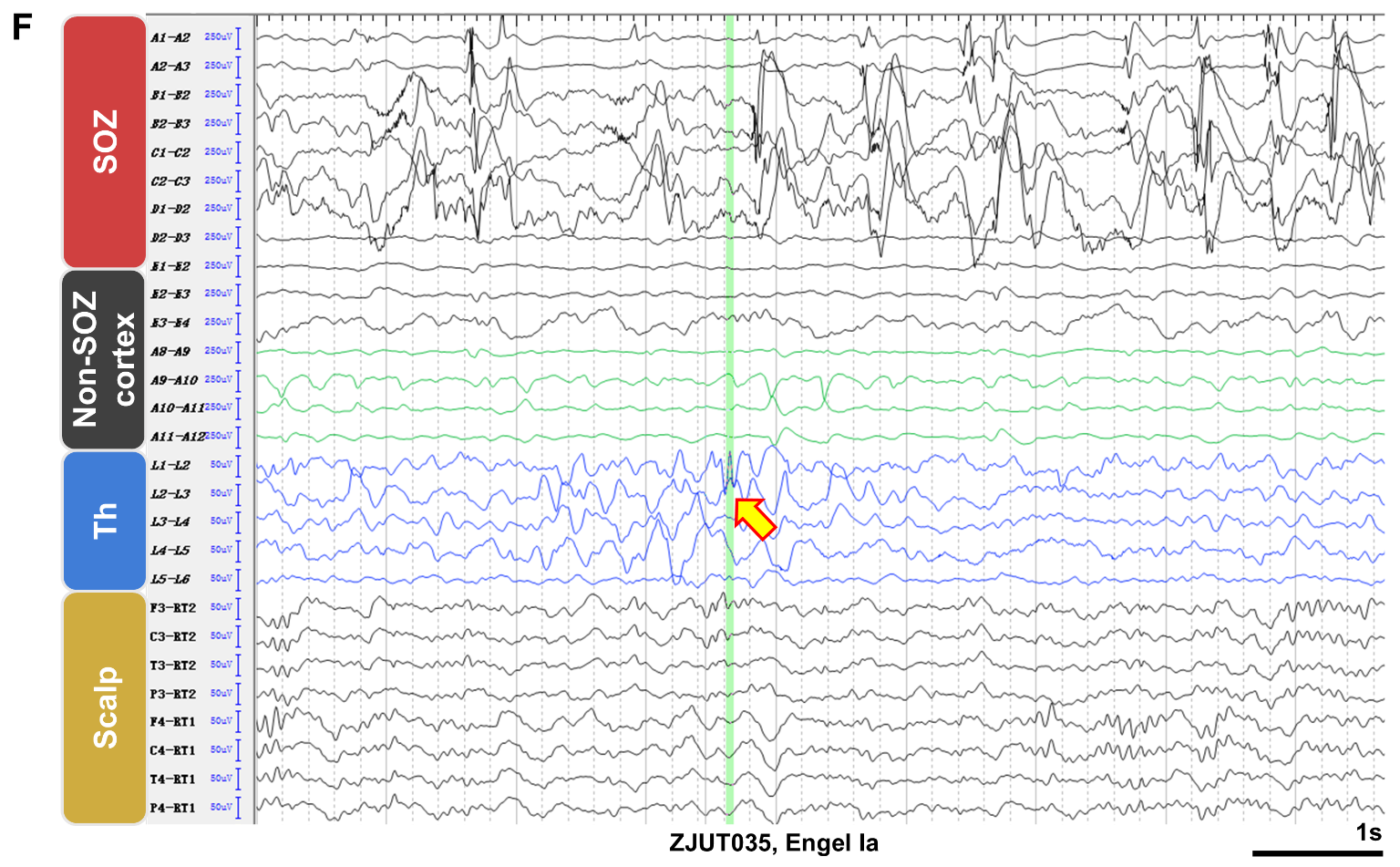


**Figure S12**. Examples of isolated spikes in the thalamus. Intracranial signals were filtered between 0.2 and 250 Hz, and scalp signals were filtered between 0.2 and 70 Hz. Patient numbers and surgical outcomes are presented at the bottom. The "RT" channel in the scalp is located in the mastoid. A: The SOZ of this patient was located in the mesial temporal lobe, and the pathology was hippocampal sclerosis. B: The SOZ of this patient was located in the anterior cingulate cortex, and the pathology was gliosis. C: The SOZ of this patient was located in the anterior insula and operculum, and the pathology was FCD IIa. D: The SOZ of this patient was located in the mesial frontal lobe, and the pathology was FCD IIb. E: The SOZ of this patient was located in the posterior insula and operculum, and the pathology was FCD II. F: The SOZ of this patient was located in the mesial temporal lobe, and the pathology was gliosis. SOZ, seizure onset zone; Th, thalamus; FCD, focal cortical dysplasia.


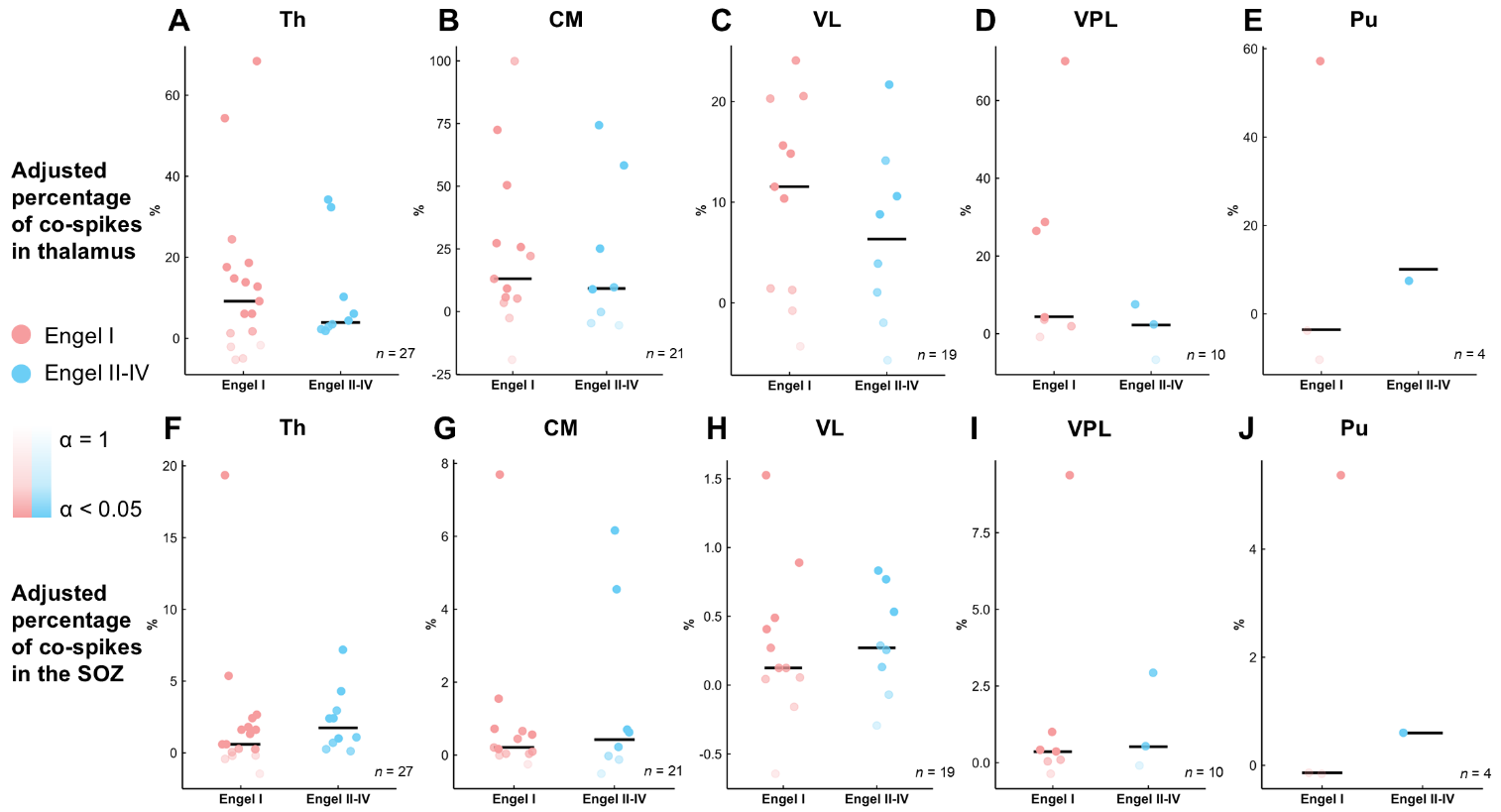


**Figure S13.** The proportions of co-spikes between the SOZ and thalamus in patients with focal SOZs and their relationships with surgical outcomes when using the raw output from the automated spike detector. A–E: The percentage of co-spikes obtained when the number of thalamic spikes was used as the denominator. F–J: The percentage of co-spikes obtained when the number of SOZ spikes was used as the denominator. Each dot represents one subject. The transparency of the colour indicates the alpha value in the surrogate data analysis. Lower transparency means that the number of co-spikes is higher than the chance level. Statistical test: Mann–Whitney U test. SOZ, seizure onset zone; Th, thalamus as a whole; CM, centromedian nucleus; Pu, pulvinar nuclei; VL, ventral lateral nucleus; VPL, ventral posterolateral nucleus.


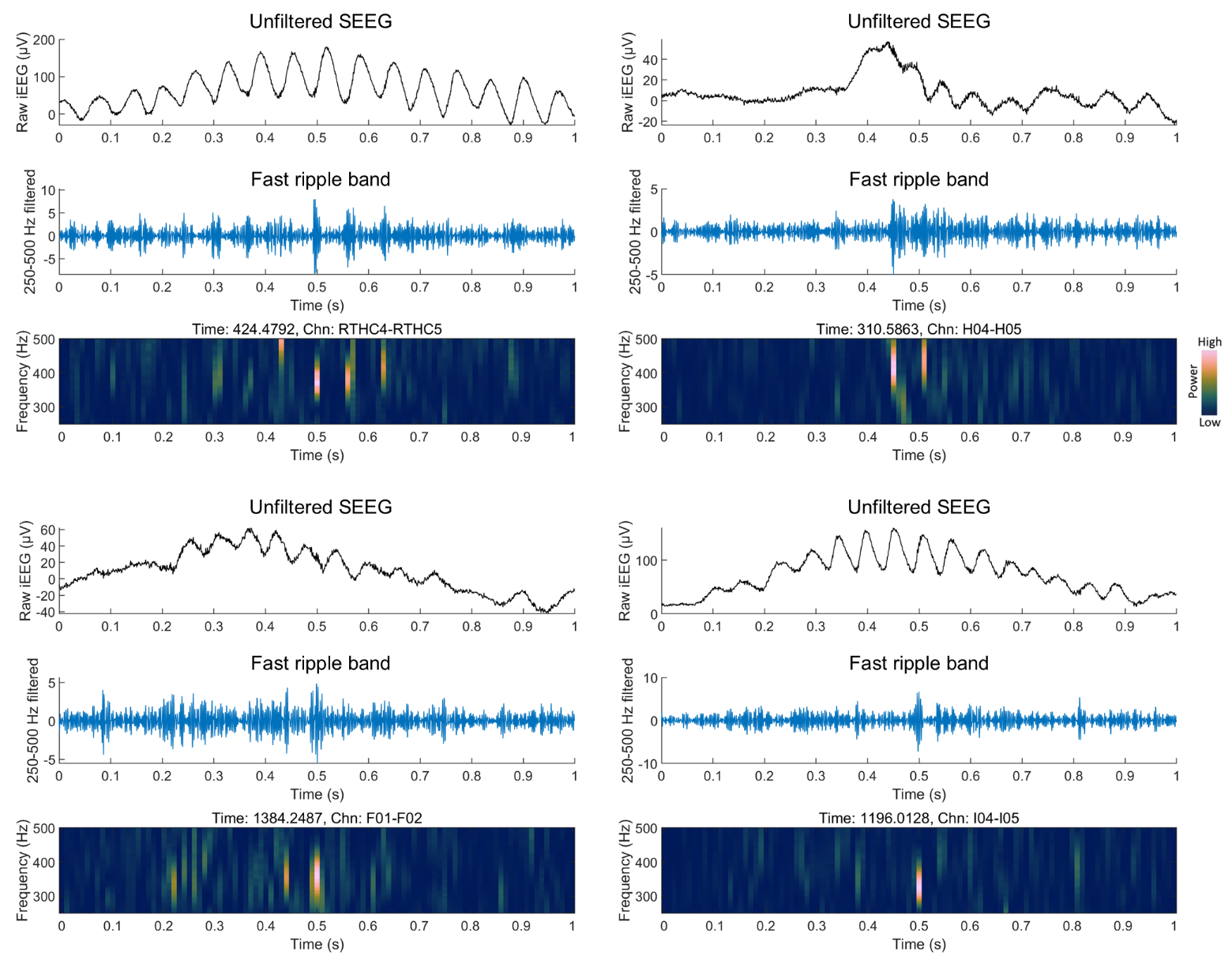


**Figure S14.** Examples of fast ripples superimposed on sleep spindles in the human thalamus. Signals and time-frequency spectra are showed in the figure. SEEG, stereo-electroencephalography; Chn, channel number.
